# Orgasmic birth: the anatomy of pleasure in childbirth

**DOI:** 10.3389/fgwh.2025.1565300

**Published:** 2025-05-13

**Authors:** Debra Pascali-Bonaro

**Affiliations:** Orgasmic Birth, Asbury Park, NJ, United States

**Keywords:** orgasmic birth, pleasurable births, respectful maternity care, sexuality, childbirth, birth hormones, the anatomy of pleasure, birthgasm

## Abstract

Contemporary childbirth practices, which have long been globally dominated by a biomedicalized framework, have sterilized the birthing experience, have stripped away emotional and physical pleasures as well as essential aspects of women's sexuality, and have led to a loss of autonomy for many women and birthing people. In this article, I propose an alternative model of care— “orgasmic birth”. And I explain that this model emphasizes respectful perinatal care, pleasurable births, and childbearers’ empowerment. By drawing on my reviews of the extensive scientific literature on hormonal interplays, the anatomies of pleasure, and sexuality, in this article, I also discuss how biomedical interventions such as synthetic hormones, epidural nerve blocks, and cesareans disrupt the natural hormonal flow that is associated with pleasurable births. The results of my literature reviews have shown that a more holistic, respectful approach to perinatal care can reduce pain, decrease birth traumas, and improve satisfaction by acknowledging the important roles of pleasure and sexuality during childbirth. I also strongly argue that future research should explore how biomedical systems can integrate practices that honor both safety and the potential for pleasure during childbirth.

## Introduction

My great-grandmother Angelina had repeatedly described having given birth to eight children at home in the early 1900s. She spoke of challenge and intensity but had never mentioned pain or fear. Her stories illustrated how she had freely walked, moved, and gave birth in upright positions, which have long been proven to facilitate the birthing process [for examples of the multiple benefits of using such positions, see (1–3)] Observing her siblings’ being born had given Angelina's oldest daughter—my grandmother Filomena—confidence in her own ability to give birth. However, by her first pregnancy, what is now called the “biomedical and/or the technocratic model of birth” had become hegemonic [for full descriptions of this model, see (4, 5)]. She had lacked the support systems and female companionship from which her mother's births had benefited. Filomena recalled feeling alone and scared, guided not by her body's instincts but by a male doctor who had given her instructions to stay in bed during her labors and births. Her story illustrated pain, fear, and the silencing of her voice and of her inner wisdom. In just one generation, the birthing paradigm had been transformed from a model of woman-centeredness to a paradigm of biomedical hegemony.

Inspired by my great-grandmother's birth stories as joyful and pleasurable, I have dedicated my career to not only having served as a doula and a doula trainer for many years, but also by evaluating the factors that contribute to birthing people having positive birth experiences. My approach to childbirth, which I have long called “Orgasmic Birth”, disputes the dominant cultural narrative that childbirth is inherently painful and traumatic. As early as 1955, Jessie Shirley Bernard and Niles Newton had described women's behaviors during their births as “sexual”. Some researchers have noticed that laboring women exhibit similar expressions and behaviors to those experienced during moments of sexual intimacy, including “breathing patterns and vocalizations [and a] loss of social inhibitions”. (ibid. pp 87–88).

Bernard and Newton (ibid. p 217) had also compared birth with an orgasm and had additionally shown that birthing women often have an “unusual strength and agility at delivery and orgasm; a sudden return of awareness afterwards; and feelings of elation, joy and well-being following both experiences”. During their labors, women's natural physiology lets them experience pleasure “irrespective of the presence of other experiences such as pain” (ibid. p 39), which, as researcher Elisabeth Bolaza (6) has importantly found, can aid in fostering positive memories and can help women to have better and more pleasurable labor and birth experiences.

Understanding the hormones of pleasure, sexuality, and childbirth has shed light on Niles Newton's (1955) observations of their multiple benefits. The hormones of childbirth (prolactin, oxytocin, adrenaline, beta-endorphins) flow similarly to those of orgasms and can be disturbed and interrupted by the usually misguided and often unnecessary biomedical interventions in the birthing process [for multiple examples of these unnecessary and longstanding interventions, see (3, 5, 7–9); among many others]. Many childbirth experts have long been asking why the sexuality of labor and birth remains unacknowledged by biomedical practitioners [Buckley cited in (10, 11)].

Some women have characterized their birthing experiences as “orgasmic”; “some explicitly stated that they had an orgasm at the moment of delivery” [Buckley cited in (10), p 216]. As Elizabeth Davis and I (12) have explained, to make birthing people comfortable and empowered during their labors, they and/or their partners can cultivate a safe, respectful, private environment in which both feel connected and able to exercise their sexuality.

I strongly advocate for a birth culture that respects and supports the normal physiologic birthing process, transforms “pain” to “power”, and incorporates moments of pleasure, joy, and love during pregnancies, labors, and births. To achieve this type of birth culture, I believe that it is important for birthing people to understand how the female anatomies of reproduction and sexuality can provide opportunities for healing, comfort and—in optimal heightened states—orgasmic feelings that may include experiences of having what Mayberry and Daniel (13) have called a “birthgasm”—an important term that I have adopted for my own use. As some researchers [for a few examples, see (2, 3, 5)] have shown, many aspects of standard childbirth care, such as the appearance of labor and delivery rooms as “sickrooms”, have negatively affected laboring people's comfort and their birth outcomes. To find pleasure during births, a sensuous environment that creates a feeling of safety and privacy is needed. My experiences of having attended hundreds of births as a doula have shown that while it is usually easier to create feelings of safety and comfort during home births and births in freestanding birth centers, doulas and midwives—like lifeguards on a beach—provide a gentle and supportive presence for women and their partners during their hospital births.

## Discussion

### Pleasure: the missing element of childbirth education

My experiences have also shown that pleasure aids healing and should be included in preparation for childbirth, as it has calming effects, aids in pain management, and creates heightened states of ecstasy. The first birth pleasure study had found that pleasure during childbirth had “included emotional, mental, spiritual, physical, and sexual aspects” [(6) p 5]. The types of pleasure that Bolaza had most frequently cited had been emotional (98.9%), mental (91.8%), spiritual (76.9%), physical (61.3%), and sexual pleasures (14.5%) (ibid.). Bolaza had also found that, for those who had chosen to give birth in their homes, their pleasure experiences had been heightened.

Women give birth through their sexual organs—the cervix, vagina, labia, and clitoral complex, yet standard biomedical childbirth practices and biomedical practitioners do not typically acknowledge the strong connections between birth and sexuality. Having been constrained by cultural taboos, even biomedical texts omit the clitoris, which, as Rachel E. Gross (14) has importantly noted, is biomedicine's most neglected human organ. I firmly believe that this lack of sexual awareness jeopardizes the survivors of sexual abuse, thereby leaving them unprepared and without having opportunities to access healing and knowledge, and also thereby potentially exacerbating their traumas instead of helping them to have healing childbirth experiences. I have also found that understanding one's pleasure anatomy and learning about the important role of the clitoris in childbirth provides an opportunity for many women who are not familiar with their clitoral complex to expand their pleasures during their pregnancies and to learn ways to use pleasure as a comfort measure during their childbirths.

During a prenatal appointment, a client profoundly shifted my perspective. When I asked if there was anything else she wanted me to know to support her, she replied, “I will be making many sounds”. Smiling, I explained that women often enter a liminal space, making primal, unique sounds during birth. She insisted, “You haven't heard mine”, demonstrating a deep Mmmmmm vibration. Opening a drawer, she revealed vibrators gifted by friends to prepare for an orgasmic birth.

During her labor, I watched her experience ecstasy during each surge, as she put the vibrator on her clitoris and felt pleasure instead of feeling pain. When she had wanted to enter the birthing pool, but was troubled because she didn't have a waterproof vibrator, my job as her doula had become wrapping vibrators in plastic wrap so she could enjoy both being in the water and her pleasurable vibrations. It had been obvious to her midwife and to me that this revelation should be widely shared with those who wish to have orgasmic births. We had realized that we too had been constrained not to think outside the biomedical birthing paradigm [for full descriptions of this paradigm, see (4, 5)]. We had asked each other: “Why was this not a commonly discussed non-pharmacological pain reduction technique?” It had seemed to me that everyone would want to know about how masturbation and vibrators could ease pain and could provide pleasurable waves. Many other clients who had experienced pleasure during their births had shared feelings of expansion and of Oneness with the Universe—a powerful feeling of divine female energy.

Many couples find that kissing, nipple stimulation, clitoral stimulation, and other intimate acts provide sensations of pleasure during their labors and births [(15), p 233]. Nevertheless, societal taboos have prevented the adoption of pleasure practices during biomedicalized childbirths. I strongly insist that it is time to “rEvolutionize” (footnote) childbirth practices, to understand birth as a part of a woman's sexuality, and to foster an environment wherein respect, safety, sensuality, and love converge to facilitate a pleasurable and orgasmic birthing experience.

### Defining orgasmic birth

Various researchers, including (6, 13, 14, 16, 17), have shown that “Orgasmic Births” can elicit positive outcomes for women and birthing people by ensuring that expectant parents have access to safe, respectful, positive, pleasurable births. For me, Orgasmic Birth involves creating positive language about birth, women's bodies, and their sexuality—finding moments of pleasure and feeling an inherent power. In our book *Orgasmic Birth: Your Guide to a Safe, Satisfying Birth Experience*, midwife Elizabeth Davis and I had described orgasmic birth as “broad enough to include those who describe birth as ecstatic and specific enough to give voice to those who actually feel the contractions of orgasm and climax” [(12), p xi.].

In my Orgasmic Birth Practitioner Program, I teach birth doulas, midwives and other healthcare professionals how to integrate pleasure practices into their work. The interconnected nature of sexuality and childbirth urges both birth activists and biomedical practitioners to reevaluate the currently hegemonic model of biomedical care and of childbirth preparation. I additionally believe that, by reframing childbirth as a pleasurable and sensual life event, birthing people can have their agency restored and can become empowered by their pleasurable experiences during their pregnancies, births, and postpartum periods.

### Birth hormones, reproductive physiology, and sexuality in labor

Oxytocin is often called “the cuddle hormone” and “the hormone of love” because it provides feelings of intimacy, relaxation, and connection. In other words, boosting oxytocin during labors supports the normal physiology of childbirth and also supports the MotherBaby bond [for multiple examples of the importance of supporting this bond, see (11, 18–20)]. I have importantly noted that there are many natural ways to increase oxytocin during childbirth, including eye-to-eye contact, loving words, safe touch, massages, and long hugs. To these other techniques, the National Partnership for Women and Families (21) has added:

staying calm, comfortable and confident, avoiding disturbances, such as unwelcome people or noise and uncomfortable procedures, staying upright and using gravity so your baby is pressed against your cervix and then, as the baby is born, against the tissues of your pelvic floor. Stimulating your nipples or clitoris before birth, and giving your baby a chance to suckle (breastfeed) shortly after birth and avoiding epidural analgesia.

A great deal of research has demonstrated the very important role of sexuality during childbirth and has described the roles of oxytocin and beta-endorphins in providing comfort and pleasure to birthing people [for multiple examples of this important role, see (6, 11, 12, 17, 22, 25)]. And as Robbie Davis-Floyd (5), pp 118–119 has clearly demonstrated, biomedical practitioners are often more comfortable with epidurals (despite their multiple negative effects on the baby [ibid.) because they don't have to provide much support to anesthetized birthing people and therefore are generally reluctant to explore alternative pain relief measures, such as masturbation, that draw on childbearers’ sexuality.

Similarly, as Margaret Jowitt (16) and Rachel Gross (14) have explained, the clitoris, which has 8,000–10,000 nerve endings, is rarely mentioned in obstetrical texts and in childbirth education classes. And as midwife Robin Lim (22, p 18) has described: “During labor, as the baby's head [or butt or feet if (the baby) is (in a breech position)] descends through the pelvis, into the vagina, eventually pressing upon the pelvic floor, the clitoris of the mother becomes activated”. A great deal of research has additionally found that using upright and/or hands-and-knees (all fours) birthing positions can impact the pressure imposed on the clitoris and can promote the release of oxytocin, which is ideal for pain relief [for multiple examples of the many benefits of using these positions, see (1, 2, 5, 9)]. For example, the back lying (lithotomy) position, which, as Robbie Davis-Floyd (5, pp 137–139) has also fully explained, is far too often used for the ease and comfort of biomedical practitioners. This position minimizes clitoral pressure and creates longer, harder, and more painful uterine contractions. In contrast, standing upright and leaning forward enhances the baby's ability to rotate through the mother's pelvis and creates multiple opportunities for birthing people to experience pleasure [for examples of the many benefits of using upright positions, see (1, 16).

I also find it important to explain that the interconnectedness of sexuality and birth is apparent in “the Ferguson reflex”, which is also called “the fetal ejection reflex”, and is a spontaneous, involuntary urge to push that occurs. During this reflex, the birthing person's body expels the baby, and the mother has no need to push. As a baby's head descends into the lower vagina during labor, pressure on the cervix triggers an oxytocin release. As Sarah J. Buckley (11) had explained, this hormone advances labor by making contractions stronger as well as by reducing pain sensations [see also Buckley in (10), p 221]. I additionally note that understanding the extremely important role of the clitoris during childbirth and the corresponding hormones that are released provides an opportunity to address the cultural biases surrounding female sexuality and/or the sexuality of birth. I also encourage expectant parents to create their own definition of “orgasmic birth”. My experiences have additionally shown that parents who have access to this knowledge about their bodies and about what supports their hormones to flow will be much more prepared to support their emotional wellbeing, sexuality, and pleasure during this transformative life event.

### The “birthgasm”

How often a “birthgasm” occurs remains unknown. Most of the women with whom I had spoken who had experienced a birthgasm had never told their partner, their best friend, or their midwife, doctor, or nurse about it. I am not advocating that a birthgasm should be the desired outcome for every birth, as I would not want to make it a performance standard that would constitute a failure if not achieved. Yet I additionally believe that it is very important for birthing people to know that it is possible to have a birthgasm and to be prepared to experience waves of pleasure during their births with no cultural shame being attached to these experiences. Many expectant parents do not understand the normal physiology of childbirth and how it connects to female sexuality, so I firmly insist that biomedical maternity care professionals have a responsibility to educate their clients about the pain relief that is available through clitoral stimulation (13).

As I have additionally previously mentioned, pleasure practices such as masturbation, the use of vibrators, and intimacy with a partner can be used as tools for providing comfort during childbirth. While these practices may not suit everyone, I also firmly believe that this knowledge and the opportunity for individuals to explore these options should not be withheld from anyone. And, as Moccia et al. (23) had relevantly shown, because pain and pleasure travel along the same neural pathway, pleasure can reduce pain sensations and can result in childbearers having more positive birth experiences. And, as Beverly Whipple and Barry R. Komisaruk (24) had demonstrated, pleasurable vaginal stimulations have been found to increase women's pain threshold by more than 75% and by more than 100% in those who had experienced an orgasm, which leads to the question: Why is this kind of pleasurable vaginal stimulation not commonly taught and used as a non-pharmacological form of pain relief?

### Methods for creating pleasurable births

One of my favorite ways to invite pleasure into birth is through daily pleasure practices. I ask my clients to create a list of 1–3-minute pleasures (e.g., breathing, music, dancing, touch) and practice three of these daily. Next, I ask them to identify pleasure practices for labor. I additionally ask my clients to reflect on whether they feel safe discussing pleasure, sexuality, and if they wish to create a private, intimate environment with their birth team. I also explain to my clients that emotional barriers can prevent them from having pleasurable births.

### Conclusion: “rEvolutionizing” the birthing domain

Here, I reiterate that the interconnected nature of sexuality and childbirth urges both birth activists and biomedical practitioners to reevaluate our current biomedical models of care and childbirth preparation. I also firmly believe that, by reframing childbirths as pleasurable and as sensual life events, women and birthing people can have their agencies restored and can also become empowered by their pleasurable birthing experiences! As I have also previously noted, this reframing requires removing cultural taboos and shame and replacing them with scientifically evidence-based practices that affirm the power of pleasure. I reaffirm that it is time to “rEvolutionize” childbirth practices, to understand birth as a part of a woman's sexuality, and to foster environments wherein respect, safety, sensuality, and love converge to facilitate pleasurable and orgasmic birthing experiences. I also strongly believe that focusing on bringing pleasure into births can reduce birthing people's experiences of pain, fear, and violence. And I conclude this article by again noting that biomedical professionals have the science and knowledge that can help them to make birth safer, respectful, and more pleasurable, and that now they need the will and the education to do so!^1^

## Data Availability

The original contributions presented in the study are included in the article/Supplementary Material, further inquiries can be directed to the corresponding author.

## Footnotes

^1^A term that I had invented because, during the process of human evolution, as Wenda Trevathan (9) had clearly explained, birthing women had almost always been assisted by their local midwives.
